# Changes in saliva composition during different training phases in professional football players

**DOI:** 10.3389/fdmed.2026.1869353

**Published:** 2026-08-05

**Authors:** Antoon J. M. Ligtenberg, Ektor C. Petras, Kamran Nazmi, Henny Solleveld, Marja L. Laine, Sergio Bizzarro

**Affiliations:** 1 Department of Oral Biochemistry, Academic Centre for Dentistry Amsterdam (ACTA) University of Amsterdam (UvA) and Vrije Universiteit Amsterdam (VU), Amsterdam, Netherlands; 2 Department of Periodontology, Academic Centre for Dentistry Amsterdam (ACTA) University of Amsterdam (UvA) and Vrije Universiteit Amsterdam (VU), Amsterdam, Netherlands; 3 SportsInjuryLab, Dronten, Netherlands

**Keywords:** diagnostics, exercise, exertion, overtraining, saliva

## Abstract

**Objective:**

This study investigated changes in salivary biomarkers collected by oral rinsing during different phases of a standard professional football training, and how these changes correlated with perceived training intensity.

**Methods:**

This study included 28 professional football players. Saliva was collected by means of oral rinse with water. It was collected at three time-points during a single training session: before training, after warming-up, and after outdoor training. Players were asked to self-report the intensity of the training after each time-point on a scale from 1 to 10. The following biomarkers were measured: protein, carbohydrate and immunoglobulin-A (IgA) concentrations, and enzymatic activity of *α*-amylase, lactoperoxidase and lysozyme.

**Results:**

Protein concentration increased during training (*p* < 0.001). *α*-Amylase activity/protein ratio (*p* < 0.001) increased while the IgA/protein ratio(*p* < 0.001) and carbohydrates/protein ratio (*p* = 0.001) significantly decreased. No association was found between the self-perceived intensity of training and changes in the biomarkers.

**Conclusions:**

This study shows that saliva composition changes in response to a regular football training session. These changes did not correlate with self-perceived intensity of training.

## 1 Introduction

Football is one of the most popular sports worldwide. In the Netherlands, the competitive season lasts nine months, during which players are expected to perform optimally. The busy match schedule combined with intensive training places a significant physical burden on players, leading to the risk of injuries and overtraining. This poses a challenge for players and coaching staff. Training must provide sufficient stimulus for each player to improve, while simultaneously minimizing the risk of injuries and overtraining. Team training programs often fail to adequately account for individual variations in aptitude and fitness. Measuring training intensity on an individual basis can offer a solution. Parameters such as heart rate and heart rate variability are used for this purpose, and blood samples are taken to determine parameters such as lactate and cortisol (1, 2). Limitations of blood sampling are that it is invasive, may be painful, and cannot be performed in unlimited frequency. Blood sampling requires trained personnel and may induce stress, which can influence biomarker levels. Furthermore, it is less suitable for frequent or field-based measurements due to practical and cost constraints. In contrast, saliva sampling is simple, stress-free, and can be performed repeatedly during training by non-specialized staff.

Saliva contains a rich array of biomarkers including hormones, enzymes, immune proteins, and metabolites. These biomarkers provide valuable insight into stress, fatigue, hydration, immune function, and overall athlete readiness (3). In addition to its relevance in systemic monitoring, saliva plays a major role in the maintenance of oral health, contributing to functions such as digestion, lubrication, and protection against oral diseases (4).

Saliva is produced by three pairs of major salivary glands namely parotid, submandibular and sublingual glands as well as numerous minor salivary glands distributed throughout the oral mucosa. The parotid salivary gland produces serous saliva rich in amylase. The submandibular and sublingual salivary glands produce mucous saliva rich in lysozyme (5). The minor salivary glands produce very mucous saliva. In addition to glandular secretions, serum-derived proteins can enter the oral cavity through gingival crevicular fluid depending on the health of the gums. In a healthy mouth the contribution is negligible, but with gingivitis, the flow of crevicular fluid increases and can account for more than half of the protein concentration (6). Salivary secretion is regulated by the autonomic nervous system, with sympathetic stimulation primarily promoting protein secretion and parasympathetic stimulation primarily promoting fluid secretion. The various salivary glands differ in balance of their autonomic innervation. The parotid and submandibular salivary glands are innervated by both sympathetic and parasympathetic nerves, while the sublingual and minor glands receive primarily parasympathetic innervation (7).

Exercise is a strong sympathetic stimulus and leads to increased protein secretion (8, 9). Protein concentration increases significantly above the anaerobic threshold, which is why it is sometimes referred to as the saliva threshold (8). Most salivary proteins are synthesized in the salivary glands and stored in vacuoles before being secreted. Immunoglobulin A (IgA) secretion follows a different pathway. IgA is produced in plasma cells and absorbed by the salivary glands and excreted as secretory IgA into the oral cavity (10).

Saliva collection can be time-consuming and may disrupt training. Moreover, saliva collection can be challenging when saliva flow is reduced due to dehydration or when athletes are panting heavily during intense exercise (11). Therefore, saliva was collected via oral rinse. In a recent on-field experiment in professional football players, saliva collection by oral rinse was shown to be a feasible method to measure changes in composition of specific biomarkers during a regular outdoor training (12). However, the extent to which saliva composition changes during different phases of a training session has yet to be elucidated.

The aim of this study was to investigate how saliva composition changes during one single training session of a professional football team and how this relates to perceived fatigue. For this purpose oral rinse was collected at three time points: before training, after warming-up, and after training. For each time point perceived training intensity was assessed and various salivary biomarkers were analyzed. We hypothesize that different levels of training intensity have impact on saliva composition.

## 2 Materials and methods

### 2.1 Study cohort

This cohort study was approved by the Ethical committee of ACTA (research proposal 2023-40395). The study population included 28 professional football players (aged >18 years), of the same professional level, affiliated with the Dutch team in the “Eerste Divisie”, which is the second highest national level. During a typical competition week. players trained once a day on Monday, Tuesday and Wednesday, twice a day on Thursday and Friday, and they played a match on Sundays. Prior to the study all participants were provided with information about the research, and they signed an informed consent. The study was designed to minimize interference with the daily routine of the football players. Inclusion criteria required participants to be over 18 years of age, in good systemic health and willing to participate. Because of the novelty of the use of oral rinse during training, this study was considered explorative. Therefore, no prior power calculation was performed.

### 2.2 Sampling method and physical activity

The training session took place at the training centre in February 2023 from 10:00 AM to 12:30 PM, with an indoor temperature of +22 °C and an outdoor temperature of +9 °C.

Saliva was collected at three different time points during a single training session. The first saliva sampling took place in the changing room just before the warming-up began (indoor). The second saliva sampling occurred indoor immediately after completion of the warming-up. The third saliva sampling took place immediately after the end of the training session on the outdoor training field before the players came back to the changing room. The indoor warming-up lasted 30 min and included both aerobic and anaerobic training. The subsequent outdoor activities extended for 120 min, encompassing aerobic training and game simulation. Following the warming-up and outdoor training sessions, participants were asked to provide a subjective rating on a scale ranging from 0 to 10, quantifying the perceived intensity of their training at each measurement instance. A value of 0 was assigned for the first sampling moment during rest to gauge the participants’ subjective assessment of effort for the other timepoints.

The saliva was collected by means of oral rinse. On the day of sampling, participants were instructed to abstain from eating and tooth brushing for 60 min before the first sampling. Only water consumption was allowed up to 30 min before sampling. No organized exercise took place prior to the first sampling. During the training, they were specifically instructed to consume only water for rehydration. There was no monitoring of the individual water intake. At the time of collection, participants were asked to swallow thoroughly. Subsequently, each participant rinsed intensively with 10 mL of tap water for 20 s and expectorated into a 50 mL polypropylene collection tube (Greiner Bio-One, Germany). Immediately after rinsing, all samples were placed on ice and processed within 12 h. The samples underwent centrifugation at 4,000 rpm for 10 min at +4 °C using an Eppendorf centrifuge (5810R, Eppendorf AG, Hamburg, Germany). Following centrifugation, each sample was aliquoted into at least 8 aliquots of 1 mL supernatant and stored at −20 °C for laboratory analysis.

### 2.3 Analysis of salivary biomarkers

#### 2.3.1 Protein concentration

Total protein content was determined using a Pierce BCA Protein Assay Kit (Thermo Scientific, Multiskan FC, Shanghai, China) in 96-well polystyrene microplates (Greiner Bio-One, Frickenhausen, Germany) following the manufacturer’s instructions with bovine serum albumin (BSA, 0–1.5 mg/mL) serving as the standard (13).

#### 2.3.2 Carbohydrate concentration

Carbohydrate concentration was determined as described previously (14). 25 µL oral rinse was mixed with 75 µL 0.1% sodium meta periodate, freshly prepared in 10% acetic acid, in non-affinity microplates (Greiner) and incubated for 2 h at 37 °C. Hereafter, 100 µL Schiff's reagent (Merck, Darmstadt, Germany) was added and incubated for 1 h at 37 °C. Hereafter, colour was measured at 570 nm with a microplate reader (Multiskan FC, Thermo Scientific, Breda, Netherlands). N-acetyl galactosamine (0–2 mg/mL) was used as a standard.

#### 2.3.3 Secretory IgA

Secretory IgA was quantified by an enzyme-linked immunosorbent assay (ELISA). High affinity microplates (Greiner BioOne) were coated with rabbit anti-human secretory IgA (Sigma-Aldrich) overnight at 4 °C. Subsequently, microplates were incubated with saliva samples and a standard of purified human secretory IgA (0–2 µg/mL, Nordic-MUbio, Susteren, the Netherlands) for 2 h at 37 °C. Detection of captured secretory IgA was achieved using HRP-conjugated goat anti-human IgA) and Sigmafast-OPD as staining solution (Sigma-Aldrich) (13).

#### 2.3.4 *α*-Amylase activity

10 μL oral rinse was diluted with 30 μL of HPLC water. Subsequently, 50 μL of 2-chloro-4-nitrophenyl-α-D-maltotrioside (CNPG3) was added as a substrate, subject to degradation by amylase into 2-chloro-nitrophenol (CNP) and maltotriose (15). The colour development of CNP was monitored at 405 nm using a microplate reader (Thermo Scientific) every minute for 10 min, serving as a measure for amylase activity. A standard of 1 U/mL human amylase (Sigma-Aldrich) was employed, and amylase activity was calculated using the formula (*Δ*abs rinse/*Δ*abs standard).

#### 2.3.5 Lysozyme activity

Black 96-well polypropylene microplates (Greiner Bio-One) were employed in all fluorescence-based enzymatic activity assays. Lysozyme activity was determined using an EnzChek Lysozyme Activity kit (Thermo Scientific), adhering to the manufacturer's guidelines (13).

#### 2.3.6 Lactoperoxidase

Lactoperoxidase activity was assessed by measuring conversion of pyrogallol to purpurogallin as described previously (16). Bovine lactoperoxidase (5 ng/mL in 100 mM potassium phosphate buffer, pH 6.0) was used as a standard. Oral rinse was mixed 1:1 with 200 mM potassium phosphate buffer pH 6.0. To a non-affinity microplate (Greiner Bio-One) 80 µL oral rinse solution, 100 mM potassium phosphate solution or lactoperoxidase solution was added. Substrate was prepared by mixing a 0.5% H_2_O_2_ solution with a 5% pyrogallol (Sigma, USA) solution in a 1:5 volume ratio. Subsequently, 120 µL substrate solution was added to each well bringing the final volume to 200 µL per well. The oxidation of pyrogallol to purpurogallin was monitored at 405 nm using a microplatereader. Measurements were taken immediately after substrate addition and continued for 5 min to monitor the enzyme activity.

### 2.4 Statistical analyses

Statistical analysis was performed with SPSS software (v 26.0, SPSS; IBM Statistics, USA). Normal distribution was assessed using the Shapiro–Wilk test. As not all biochemical variables were normally distributed, the Friedman non-parametric test for repeated measures was employed to examine potential differences between time points for both the biochemical variables. Effect size was calculated with Kendall's W. Bonferroni correction was used for multiple testing between different samples time-points. Additionally, univariate analysis of co-variance (ANCOVA) was utilized to explore potential association between self-reported intensity and the differences in the biochemical variables across different time points. *P*-values ≤0.05 were considered statistically significant.

## 3 Results

### 3.1 Characteristics of the study cohort

All players were males between 20 and 37 years old with a median of 23. Average physical effort was reported as being 0 before training, 2.19 (SD 1.37) after warming-up and 5.25 (SD 1.99) after outdoor training with an overall statistically significant difference (*p* < 0.001).

### 3.2 Salivary biomarkers changes

Table 1 presents the concentrations or activities of the biomarkers in oral rinses at the three sampling moments. Mean protein concentration increased from 126.14 µg/mL at time-point 0 (T0) to 214.46 µg/mL at time-point 2 (T2)(*p* < 0.001, r = 0.39). The median protein concentration for T2 was significantly higher than at T0 (*p* < 0.001) and T1(*p* = 0.033). T1 vs. T0 was not statistically significant (*p* = 0.098). Similarly, *α*-amylase activity increased from 4.66 u/mL at T0 to 14.18 at T2 (*p* < 0.001, r = 0.46). The median *α*-amylase activities at T2 (*p* < 0.001) and T1 (*p* = 0.002) were higher than at T0, but not statistically different between T2 and T1 (*p* = 0.425). Lysozyme increased from 0.62 u/mL at T0 to 0.93 u/mL at T2 (*p* = 0.001, r = 0.25). The median lysozyme activity at T2 and T1 were significantly higher than T0 (*p* = 0.004 for both time points) but no significant difference was found for T2 vs. T1 (*p* = 1.0). Finally, lactoperoxidase increased from 11.87 u/mL to 27.21 u/mL at T2 (*p* < 0.001, r = 0.46). The median lactoperoxidase at T2 was higher than T1 (*p* = 0.033) and T0 (*p* < 0.001), and T1 was higher than T0 (T = 0.033). No statistically significant changes were found for IgA (*p* = 0.898) and carbohydrates (0.427).

**Table 1:** Absolute values of the salivary biomarkers of pre warming-up, after warming-up and after training sample time points (*N* = 28).

Salivary biomarker	T0 Mean (SD) Median	T1 Mean (SD) Median	T2 Mean (SD) Median	Overall *p*-value	Effect size(r)
Amylase (u/mL)	4.66 (3.98) 3.47	8.93 (7.96) 5.64	14.18 (12.15)^a^ 10.75	< 0.001	0.46
Lysozyme (u/mL)	0.62 (0.38) 0.53	0.86 (0.56) 0.74	0.93 (0.56)^b^ 0.85	0.001	0.25
sIgA (μg/mL)	1.00 (0.26) 1.02	0.97 (0.25) 1.06	0.96 (0.27) 1.05	0.898	<0.01
Lactoperoxidase (u/mL)	11.87 (5.34) 10.76	16.97 (8.82) 13.60	27.21 (16.53)^c^ 26.56	<0.001	0.46
Carbohydrate concentration (mg/mL)	1.63 (0.58) 1.63	1.67 (0.58) 1.67	1.68 (0.53) 1.62	0.427	0.03
Protein concentration (µg/mL)	126.14 (68.17) 117.22	168.06 (77.95) 160.67	214.46 (97.17)^d^ 201.24	<0.001	0.39

Sampling moments: T0, before warming-up; T1, after warming-up; T2, after training.

Overall *p*-values: Friedman test. Effect size (r): Kendall's W.

Post hoc Bonferroni correction:

a significant difference T1 vs. T0 (*p* = 0.002); T2 vs. T0 (*p* < 0.001).

b significant difference T1 vs. T0 (*p* = 0.004); T2 vs. T0 (*p* = 0.004).

c significant difference T1 vs. T0 (*p* = 0.033); T2 vs. T1 (*p* = 0.033); T2 vs. T0 (*p* < 0.001).

d significant difference T2 vs. T1 (*p* = 0.033); T2 vs. T0 (*p* < 0.001).

### 3.3 Relative concentration of salivary biomarkers

Since absolute concentrations in oral rinse may vary depending on the method of rinsing Table 2 presents the concentration or activity of the salivary biomarkers relative to the total protein concentration (mg/mg or u/mg). The ratio *α*-amylase/total protein increased by 21.17 u/mg during training (*p* < 0.001, r = 0.24) with a significant difference between T0-T1 (*p* = 0.048) and between T0-T2 (*p* < 0.001), but not between T1-T2 (*p* = 0.687). The ratio sIgA/total protein decreased by 3.93 μg/mg during training (*p* < 0.001, r = 0.51) with significant differences between all timepoints (T0-T1, *p* = 0.023, T0-T2, *p* < 0.001, T1-T2, *p* = 0.023). The carbohydrate/protein ratio also decreased by 6.77 mg/mg through training (*p* < 0.001, r = 0.24) with a significant difference between T0-T1 (*p* = 0.048) and T0-T2 (*p* = 0.001), but not between T1-T2 (*p* = 0.687). Ratio's of lysozyme/total protein and lactoperoxidase/total protein displayed no significant changes throughout the training (*p* = 0.565 and *p* = 0.409 respectively).

**Table 2:** Salivary biomarkers (calculated as total protein ratios) of pre warming-up, after warming-up and after training sample time points (*N* = 28).

Salivary biomarker	T0 Mean (SD) Median	T1 Mean (SD) Median	T2 Mean (SD) Median	*p*-values T0-T1^a^	*p*-values T0-T2^a^	*p*-values T1-T2^a^	Overall *p*-values^b^	Effect size (r)^b^
Amylase/ protein (u/mg)	35.81 (20.15) 35.29	46.72 (30.6) 40.55	56.98 (34.07) 60.29	0.048	0.001	0.678	<0.001	0.24
Lysozym/ protein (u/mg)	5.61 (3.31) 5.26	5.44 (3.16) 4.64	4.58 (2.62) 4.04	N.A.	N.A.	N.A.	0.565	0.02
IgA/ protein (μg/mg)	9.53 (4.15) 9.63	7.44 (5.21) 6.70	5.60 (3.30) 4.48	0.023	<0.001	0.023	<0.001	0.51
Lactoperoxidase/ protein (u/mg)	105.22 (43.34) 106.03	106.06 (39.70) 101.21	127.47 (52.57) 114.46	N.A.	N.A.	N.A.	0.409	0.03
Carbohydrates/ protein (mg/mg)	16.86 (11.52) 13.67	11.99 (6.02) 10.75	10.09 (6.95) 8.68	0.048	0.001	0.687	<0.001	0.24

Sampling moments: T0, before warming-up; T1, after warming-up; T2, after training.

a p-value adjusted with Bonferroni correction for multiple comparison.

b Overall *p*-value calculated with Friedman's test.

NA, Not applicable; IgA, Immunoglobulin-A.

### 3.4 Self-reported training intensity and their influence on salivary biomarkers

For the biomarkers that showed significant changes in response to training it was investigated whether these changes correlated with self-reported training intensity. At T = 1 significant correlation was found between self-reported training intensity and changes in lactoperoxidase activity per mL (*p* = 0.012, r = 0.470). No significant correlations were found for the other biomarkers, both expressed as changes in absolute and relative concentrations.

## 4 Discussion

The aim of this study was to investigate changes in salivary biomarkers during different phases of a professional football training and whether these changes correlated with the perceived training intensity. The results demonstrated that training affected salivary biomarkers with a clear increase in total protein concentration during the training. Among the proteins investigated *α*-amylase to total protein ratio specifically increased. Conversely, the concentrations of sIgA and the carbohydrate did not change resulting in significant decreases for their protein ratio. Lysozyme and lactoperoxidase levels followed the changes in protein concentration. The reported changes in salivary biomarkers showed no correlation with the self-reported training intensity as was found previously (17). Interestingly, we noticed that also a mild exercise (specifically a warming-up in the current study) can influence the composition of saliva, although, as expected, the major differences were found after the intensive outdoor session.

In the present study, instead of whole saliva collection by drooling or expectoration saliva was collected by oral rinsing, because this was easier and faster. This made it suitable during training where the players had to switch quickly between exercises. Collection of whole saliva during exercise not only disrupts training, but it can also be challenging as heavy breathing during intense exercise and dehydration result in dry mouth. After extreme endurance events such as a marathon some subjects could not produce saliva at all (11). A previous on-field experiment with oral rinse in football players showed that oral rinse is suitable to detect changes in saliva composition after intensive outdoor training (12). In addition, Al Habobe et al. (2024) showed that oral rinsing is a promising method for the qualitative assessments of saliva when compared with other saliva collection methods (18).

Protein concentration increased in oral rinse as was found previously for whole saliva (8, 9). Protein concentrations in oral rinses were about 10-fold lower than in whole saliva. In these studies, the increase in protein concentration was not due to a reduced saliva flow rate, but the result of an increased protein secretion. In oral rinse the flow rate could not be quantified making it uncertain whether this increase is the result of increased secretion. Therefore, next to the absolute concentrations, we analyzed also the ratios of the biomarkers in relation to the total protein concentration of the mouth rinse. Similar to what was found previously for saliva collected by spitting, oral rinse showed changes in composition (9).

Although the carbohydrate concentration remains relatively stable, their ratio to the protein concentration decreased when expressed in proportion. A relative increase in *α*-amylase levels was found previously. The increase in protein concentration after intense exercise was primarily caused by an increase in *α*-amylase (19). Possibly, the *α*-amylase to total protein ratio could serve as a marker for sympathetic system activity and training (20, 21). Overstimulation of the sympathetic system may lead to overtraining adversely affecting both athlete health and performance efficiency (22, 23).

Our previous study found no increase in protein concentration and amylase activity in oral rinse after an outdoor football training (12). In present study oral rinse after training was collected outdoor immediately after training. In the previous study oral rinse after the training was collected in the dressing room giving players some time to recover. In whole saliva protein concentration returned to baseline 30 min after running at maximum intensity for 10 min (9).

Levels of sIgA did not increase during the training sessions, resulting in a decrease of the sIgA to total protein ratio. The significant decrease in sIgA/protein during this training session may be attributed to the fact that sIgA is not directly synthesized in the salivary glands but rather by the plasma cells surrounding these glands. Therefore, when the salivary glands are stimulated by the sympathetic system, the increase in total protein does not necessarily lead to a proportional increase in sIgA, resulting in a decrease in the ratio (24). The last two biomarkers examined were lysozyme and lactoperoxidase, both of which play roles in the innate immune defence. In line with previous research lysozyme and lactoperoxidase were increased after exercise. A 50% increase in lysozyme concentration was found in a cohort of rowers performing a graded exercise to exhaustion (25), consistent with findings for other proteins (26, 27). Salivary peroxidase, a crucial enzyme within the salivary antioxidant system (28), increased during training as was found previously (29).

When looking at changes in salivary biomarkers only changes in lactoperoxidase activity at time point 1 correlated with perceived training intensity. An earlier study found a correlation between salivary cortisol and perceived exertion after one hour cycling (30), but another study found no correlation between cortisol and perceived exertion after high and low intensity resistance exercise (31). Rate of perceived exertion did not correlate with squat jump and countermovement jump as performance markers of fatigue (17). Increases in concentrations of protein and other biomarkers due to dehydration and heavy breathing might interfere with increases by exercise. Also normalized concentrations showed no correlation with perceived training intensity. Possibly, normalization to protein concentration is of limited value since protein concentration also changes upon exercise (8, 9). Normalization to a salivary component that is always constant independent of saliva flow rate or exercise might solve this problem. Perceived training intensity might not correlate with the actual training intensity as highly motivated athletes might underestimate the intensity.

The strengths of this study include its longitudinal design, which involved three consecutive sampling points throughout the training period. A limitation of this study is the use of mouth rinse as the sampling method making an accurate determination the saliva volume impossible. Concentrations and activities of the various biomarkers were set against the saliva protein concentration, but as the protein concentration also varies upon exercise, this parameter could only show changes in saliva composition.

## 5 Conclusions

This study shows that saliva, collected by oral rinse, changes in composition in response to both indoor and outdoor sessions during a regular football training. These changes did not correlate with self-perceived intensity of training.

## Data Availability

The raw data supporting the conclusions of this article will be made available by the authors, without undue reservation.
