# Supplementary material for: Changes in saliva composition during different training phases in professional football players
Source: Front Dent Med. 2026 Aug 5;7:1869353. doi: 10.3389/fdmed.2026.1869353 (PMC13485790; doi:10.3389/fdmed.2026.1869353)
Supplement: Supplementary file 1 [file Table1.docx]

Table S1. Correlation between changes in biomarkers and perceived intensity of training

| Correlation with changes in absolute concentrations (/mL) | | | | |
| --- | --- | --- | --- | --- |
|  | T0-T1 | | T0-T2 | |
|  | p-value | Effect  size (r) | p-value | Effect  size (r) |
| Protein concentration | 0.982 | - 0.004 | 0.840 | 0.040 |
| Amylase activity | 0.638 | -0.093 | 0.320 | 0.195 |
| Lysozyme activity | 0.181 | -0.260 | 0.791 | -0.52 |
| Lactoperoxidase activity | 0.012* | 0.470 | 0.368 | -0.177 |
| Correlations with relative concentrations (/mg protein) | | | | |
|  | T0-T1 | | T0-T2 | |
|  | p-value | Effect  size (r) | p-value | Effect  size (r) |
| Amylase activity | 0.331 | 0.191 | 0.347 | - 0.185 |
| IgA | 0.154 | -0.277 | 0.349 | 0.184 |
| Carbohydrate | 0.097 | -0.320 | 0.361 | 0.179 |
